# Highly Sensitive Graphene-Based Electrochemical Sensor for Nitrite Assay in Waters

**DOI:** 10.3390/nano13091468

**Published:** 2023-04-25

**Authors:** Florina Pogăcean, Codruţa Varodi, Lidia Măgeruşan, Stela Pruneanu

**Affiliations:** National Institute for Research and Development of Isotopic and Molecular Technologies, 67-103 Donat Street, 400293 Cluj-Napoca, Romania

**Keywords:** graphene-based sensors, electrochemical exfoliation, nitrite assay, amperometry, square wave voltammetry

## Abstract

The importance of nitrite ions has long been recognized due to their extensive use in environmental chemistry and public health. The growing use of nitrogen fertilizers and additives containing nitrite in processed food items has increased exposure and, as a result, generated concerns about potential harmful health consequences. This work presents the development of an electrochemical sensor based on graphene/glassy carbon electrode (EGr/GC) with applicability in trace level detection of nitrite in water samples. According to the structural characterization of the exfoliated material, it appears as a mixture of graphene oxide (GO; 21.53%), few-layers graphene (FLG; 73.25%) and multi-layers graphene (MLG; 5.22%) and exhibits remarkable enhanced sensing response towards nitrite compared to the bare electrode (three orders of magnitude higher). The EGr/GC sensor demonstrated a linear range between 3 × 10^−7^ and 10^−3^ M for square wave voltammetry (SWV) and between 3 × 10^−7^ and 4 × 10^−4^ M for amperometry (AMP), with a low limit of detection LOD (9.9 × 10^−8^ M). Excellent operational stability, repeatability and interference-capability were displayed by the modified electrode. Furthermore, the practical applicability of the sensor was tested in commercially available waters with excellent results.

## 1. Introduction

Due to its crucial involvement in the creation of cellular components, including nucleic acids and proteins, nitrogen is an essential and necessary element for all living organisms [1]. Recently, bioavailable nitrogen levels have been significantly impacted by human activities [2]. The extensive and widespread usage of nitrogen-based agricultural fertilizers has significantly expanded in the last years and is directly correlated with a profound effect on water pollution [3] and drinking water contamination [4]. The nitrogen cycle on earth includes the naturally occurring ionic species nitrate and nitrite [5]. Nitrite (${\mathrm{NO}}_{2}^{-}$), a common inorganic ion, is a byproduct of nitrifying bacteria in soil and water oxidizing ammonia nitrogen to nitrate. Normally, the nitrite amounts that can be typically present in water systems are low, but, occasionally, imbalances in the nitrifying process result in nitrite system accumulation. Moreover, the leakage of reactive nitrogen into the environment generates an overexposure to nitrites that endangers both ecosystems and human health [6]. On the other hand, the importance of nitrites has long been recognized in environmental chemistry and public health, since they are usually present in different pharmaceuticals and household items, widely employed in the food sector as food preservatives (E249, E250) [7] and corrosion inhibitors in industrial water [8]. Nitrite-containing foods are not consumed on a daily basis and nitrites do not accumulate in the body. However, endogenous ${\;\mathrm{NO}}_{2}^{-}$ is created via the oxidation of NO, as well as the reduction of dietary nitrate (${\mathrm{NO}}_{3}^{-}$), as a result of microorganisms in our saliva, stomach and intestines [9]. It has long been acknowledged that serious, harmful health consequences (such as respiratory distress, central nervous system problems, carcinogenic tumors, thyroid affections, genetic modifications, and even death) might occur from nitrite overexposure [10,11,12,13] High nitrite levels lead to a serious blood condition called methemoglobinemia or ‘Blue Baby Syndrome’, in which the body is deprived of oxygen, since nitrites oxidize the iron component of red blood cells (hemoglobin), causing them to lose their ability to transport oxygen [14]. In view of all of these health posing risks, the World Health Organization (WHO) has established guidelines on acceptable levels of human intake based on the lack of particular short-term health consequences (methemoglobinemia and thyroid effects). The WHO defines acceptable concentrations of nitrite in drinking water as 3 mg/L (214.2 μM) [15], while the Scientific Committee on Foods, confirmed by the Joint Food and Agriculture Organization (FAO)/WHO Expert Committee on Food Additives (JECFA), defines an acceptable daily intake of nitrite from food sources as 0.06 mg/kg body weight [16]. Consequently, monitoring the nitrite content in the environment, public health and the food industry is crucial, and the development of simple, sensitive and efficient analytical tools is essential and must be addressed. Until now, different types of spectroscopic [17,18], chromatographic [19,20,21], capillary electrophoretic [22] and electrochemical methods [23,24,25] have been developed and used for nitrite detection. Among these, due to its rapidity, sensibility and low costs, the electrochemical aproach has become one of the most popular and extensively used analytical tools [26]. To create electrochemical sensors with increased sensitivity and accuracy for nitrite detection, different nanomaterials have been embedded on electrode surfaces: nickel/PDDA/reduced graphene oxide [27], Ag–Fe_3_O_4_–graphene oxide magnetic nanocomposites [28], Fe_3_O_4_–reduced graphene oxide composite [29], metal-organic framework derived rod-like Co@carbon [30], La-based perovskite-type lanthanum aluminate nanorod-incorporated graphene oxide nanosheets [31], cobalt oxide decorated reduced graphene oxide and carbon nanotubes [32], gold–copper nanochain network [33], carbon nanotube (CNTs), chitosan and iron (II) phtalocyanine (C_32_H_16_FeN_8_) composite [34]; gold nanoparticles/chitosan/MXenes nanocomposite [35], worm-like gold nanowires and assembled carbon nanofibers–CVD graphene hybrid [36]; photochemically-made gold nanoparticles [37], or graphene nanoparticles [38]. Among these, the special properties of graphene are unequivocal and long-time recognized [39], justifying their extensive applicability and usage in sensors technology [40,41].

The novelty of this work is related to the development and applicability of a graphene-based sensor for the quantitative analysis of trace levels of nitrites in different commercially available water samples. Compared to the majority of previously reported modified electrodes used in nitrite assay, the prepared sensor has the advantage of a simple, one-step and low cost preparation method.

## 2. Materials and Methods

### 2.1. Chemicals

The chemicals used during the experiments were of analytical grade and were not further purified before usage. Graphite rods (6 mm diameter, 99.995% purity); potassium chloride (KCl, 99.98%); sodium dihydrogen phosphate (NaH_2_PO_4_, 100%) and di-sodium hydrogen phosphate anhydrous (Na_2_HPO_4_, 99.7%) were supplied by VWR Chemicals (Leuven, Belgium). Sodium acetate anhydrous (CH_3_COONa, ≥99.0%); ammonium sulphate ((NH_4_)_2_SO_4_, ≥99.0%), sodium chloride (NaCl, 99.5%); sodium carbonate anhydrous (Na_2_CO_3_, 99.3%), magnesium chloride hexahydrate (MgCl_2_∙6H_2_O, 99–102%); magnesium sulphate (MgSO_4_, 99%); magnesium nitrate hexahydrate (Mg(NO_3_)_2_∙6H_2_O, 99%) were provided by REACTIVUL Bucuresti (Bucharest, Romania). Potassium ferrocyanide K_4_[Fe(CN)_6_] and L(+)-Ascorbic acid (C_6_H_8_O_6_, AA, ≥99%) were acquired from Merck (Darmstadt, Germany). Sodium acetate anhydrous (CH_3_COONa, ≥99.0%) was purchased from ChimReactiv SRL (Bucharest, Romania). N,N-dimethylformamide (DMF) was acquired from Fluka Chemie GmbH (Buchs, Switzerland), while boric acid (H_3_BO_3_, 99%) was provided by Andra Chim SRL (Bucharest, Romania). The MQuant^®^ Nitrite Test (Merck KGaA, Darmstadt, Germany) was employed for UV-Vis investigation of nitrite solutions. Deionized water with a resistivity of at least 18.2 MΩ × cm was used to prepare all solutions.

### 2.2. Apparatus

In order to reveal the morphological characteristics of the graphene sample, we employed a Hitachi HD2700 instrument (Hitachi, Japan) equipped with a cold field emission gun (CSEG). The structural characterization of graphene was performed by X-ray powder diffraction (XRD), with a Bruker D8 Advance Diffractometer (40 kV; 0.5 mA) equipped with a LYNXEYE detector (λ = 1.5406 Å). After the deconvolution of the recorded pattern, some parameters of graphene samples were determined: the graphene crystallite size (D), the interlayer distance (d) and the number of layers (n). The graphene Raman spectrum was recorded with an NTEGRA Spectra platform, placed on a NEWPORT RS4000 optical table and equipped with a SOLAR TII confocal Raman spectrometer coupled with an Olympus IX71 microscope in two different configurations (Moscow, Russia). A Christ-Alpha 1-4 LSC freeze-drier (Martin Christ Gefriertrocknungsanlagen GmbH, Osterode am Harz, Germany) was employed for drying the sample obtained after electrochemical exfoliation of graphite rods.

For electrochemical measurements (Cyclic Voltammetry—CV; Square Wave Voltammetry—SWV; Amperometry—AMP; and Electrochemical Impedance Spectroscopy—EIS) a Potentiostat/Galvanostat instrument PGSTAT-302N (Metrohm-Autolab B.V., Utrecht, The Netherlands) coupled with a personal computer was employed. The working electrode was either bare glassy carbon electrode (3 mm diameter), or that modified with graphene. The counter electrode was a large platinum sheet (2 cm^2^ area) and the reference was an Ag/AgCl electrode.

### 2.3. Graphene Synthesis by Electrochemical Exfoliation of Graphite Rods (EGr)

In an electrochemical cell filled with the appropriate solution (0.05 M (NH_4_)_2_SO_4_ + 0.1 M H_3_BO_3_ + 0.05 M NaCl), two graphite rods (anode and cathode) were immersed and connected to the exfoliation system. The solution temperature was kept at around 10 °C with a thermostat and the time parameters for current pulse exfoliation were set before starting the graphene synthesis: current pulse duration 0.8 s; pause between two current pulses 0.2 s [42]. The exfoliation started after applying a bias of 12 V for 4 h, after which the process was stopped. The resulting black suspension was thoroughly washed with distilled water (8 L) to remove the ions superficially attached to the graphene flakes. The large graphite particles were removed by filtration with Whatman qualitative paper (white-ribbon filter). The last step was the drying, which was done by lyophilisation. The sample was then denoted as EGr.

### 2.4. Glassy-Carbon Modification with Graphene (EGr/GC)

N,N-dimethylformamide (DMF) organic solvent was selected for the dispersion of graphene sample (2 mg/mL). Due to its high boiling point (153 °C), it evaporates slowly at room temperature, allowing the graphene flakes to form a stable layer on top of the electrode. The GC electrode was covered with a total volume of 10 µL from the graphene sample in DMF and dried at room temperature for 24 h. We tested several electrodes covered with different volume (5; 8; 10; 12 and 14 µL) of graphene and measured their electrochemical signal towards NO_2_^−^ oxidation. The modification led to the increase of both faradaic and capacitive currents and the optimum amount was found to be 10 µL (see Figure S1 in Supplementary Materials). The electrochemical performances of bare GC and EGr/GC electrodes towards the nitrite electrochemical detection and quantification were tested and compared.

## 3. Results and Discussions

### 3.1. Morphological and Structural Characterization of Graphene Sample

**Figure 1:**
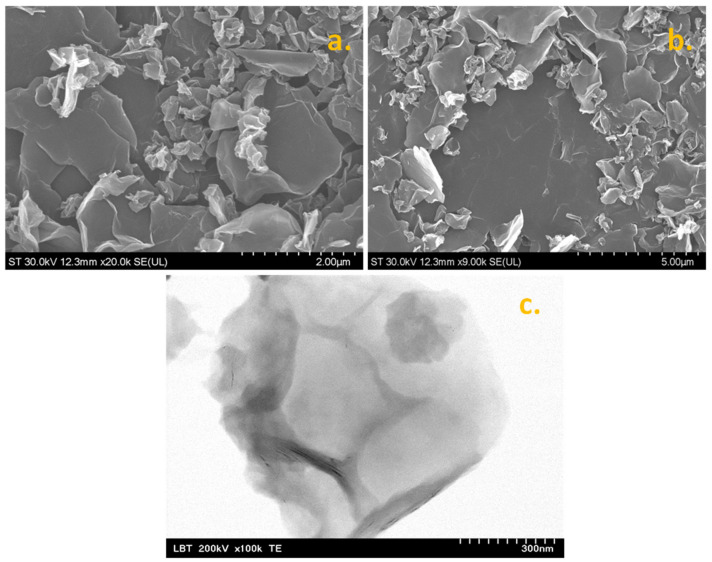
SEM micrographs of synthesized graphene powder (**a**,**b**); TEM micrograph of graphene flakes (**c**).

The morphological characteristics of graphene sample, such as the lateral dimensions of the sheets and their transparency, were investigated by SEM/TEM techniques. In Figure 1a,b, two representative SEM micrographs are shown, which clearly indicate that the sample contains flakes with lateral size ranging from hundreds of nm to a few micrometers. The basal planes of the large flakes can be visualized as grey and smooth areas, whereas the edges of the flakes appear as bright lines. The transparency of the flakes proves the successful exfoliation of graphite rods and also confirms that graphene is composed of few-layer and multi-layer sheets (see the TEM micrograph in Figure 1c). The TEM technique uses electrons that pass through the sample, so multiple sheets appear darker relative to single sheets. The flakes are not only thin but also randomly oriented, generating a porous layer when deposited on top of a solid substrate. As expected, the layer porosity increases the active area of the modified electrode, leading to an increase of the electrochemical signal. Besides the morphological aspects of graphene, the structural characteristics were also evaluated by XRD and Raman spectroscopy.

**Figure 2:**
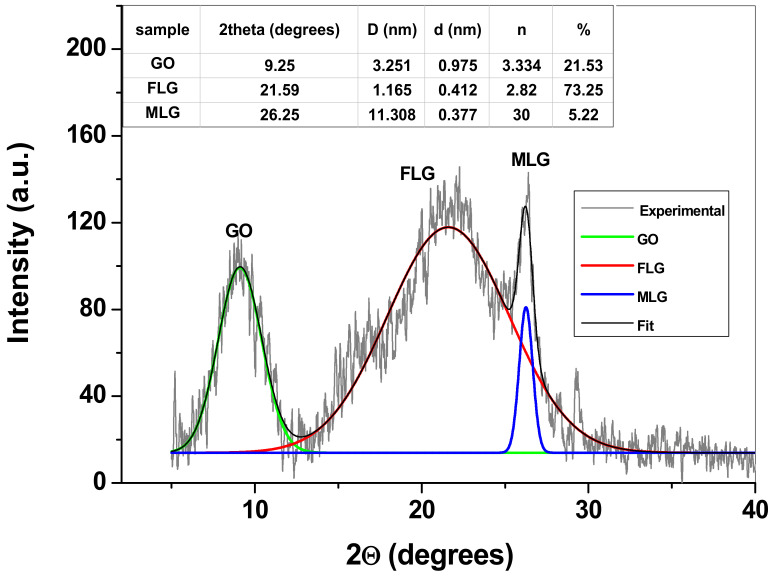
The recorded XRD pattern of graphene sample (background subtracted). *Inset table*: The structural parameters of graphene crystallites.

The XRD pattern of the sample is presented in Figure 2 and reveals three important peaks attributed to the reflections of graphene oxide (GO) layers (around 9°), few-layers graphene (FLG; around 21°), and finally multi-layers graphene (MLG; around 26°). As can be seen in the inset of Figure 2, the identified structures have different values for the *d* spacing (the distance between two adjacent layers), such as 0.975 nm for GO, 0.412 nm for FLG and 0.377 nm for MLG. The high value observed for GO may be attributed to the abundance of oxygen-containing groups attached to graphene layers, which keeps the layers apart. In the case of FLG and MLG, the number of functional groups is smaller and so is the *d* value. It is important to mention that the majority of MLG flakes were removed during the filtration procedure on Whatman qualitative paper, so their amount in the sample is small (5.22%). FLG is predominant within the sample (73.25%), while GO is 21.53%. The other structural parameters determined for graphene sample are also presented in the inset table: the mean size of graphene crystallite (D) and the average number of layers present within the graphene crystallites (n) [43].

**Figure 3:**
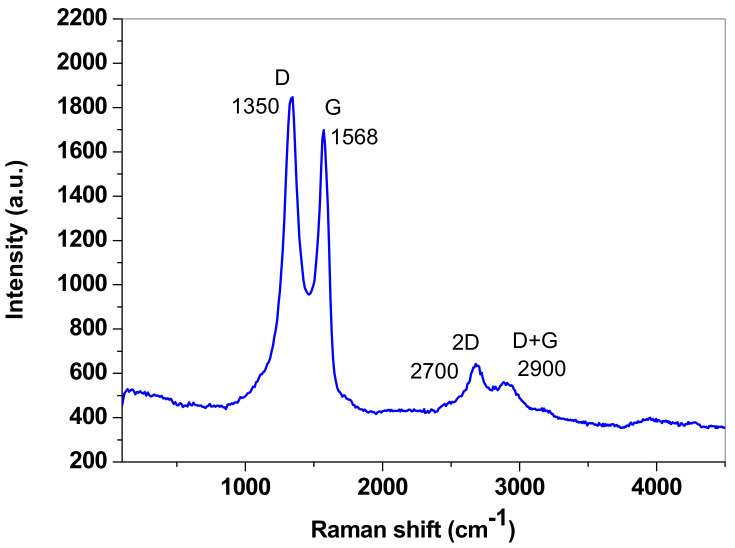
The Raman spectrum of the graphene sample.

Raman spectroscopy was additionally employed to investigate the structural disorder degree in the employed graphene sample. In Figure 3, the recorded Raman spectrum is presented, which exhibits all the characteristic bands of graphene: the defect band (D) at ~1350 cm^−1^ is the most intense and appears due to the structural defects present in the sp^2^ hybridized carbon network; the graphite band (G) at ~1568 cm^−1^ is characteristic of graphitic structures and is generated by the in-plane vibration mode of the sp^2^ hybridized carbon network; at ~2700 cm^−1^ is the 2D band, which is a second-order overtone of different in-plane vibrations; at ~2900 cm^−1^ is the D + G band, which is a combination of scattering peaks. As can be seen in Figure 3, the intensity of the D band is higher than that of the G band (I_D_/I_G_ = 1.087), which correlates well with the presence of defects in the graphene lattice. Such defects may be generated by the oxygen-containing groups from graphene oxide which, according to the XRD pattern, represents ~21.5% of the sample. According to the work of Cançado et al. [44], the I_D_/I_G_ ratio may be related to the in-plane crystallite size (L_a_) of graphene (as expressed by Equation (1)) and indicates the magnitude of the defect-free domains:

(1) $$L_{a}\left(\mathrm{nm}\right)=\frac{560}{E_{l}^{4}}{\left(\frac{I_{D}}{I_{G}}\right)}^{-1}$$

where E_l_ represents the laser excitation energy (2.33 eV).

For the synthesized graphene sample, L_a_ was determined to be 17.46 nm.

### 3.2. Electrochemical Studies with GC and EGr/GC Electrodes

Before the investigation of the electro-catalytic properties of EGr/GC electrode towards NO_2_^−^ oxidation, the active area of the graphene-modified electrode was determined and compared with that of the bare GC electrode. Hence, cyclic voltammograms were recorded with different scanning rates (from 2 to 100 mV/s) in the presence of 10^−3^ M K_4_[Fe(CN)_6_] (0.2 M KCl supporting electrolyte). Using the Randles–Ševcik equation [45] and, respectively, the I_p_ versus υ^1/2^ plot, the active area was calculated (see Figure S2a,b in the Supplementary Materials). The I_p_ versus υ^1/2^ plot was fit by the following linear regression equation: I_p_ = 2.11 × 10^−7^ + 3.11 × 10^−5^ × υ^1/2^ (R^2^ = 0.996) and, from the corresponding slope, the EGr/GC area was determined to be 0.049 cm^2^. As expected, the value was considerably larger than that obtained for bare GC electrode (0.028 cm^2^), due to the porous morphology of the graphene layer.

**Figure 4:**
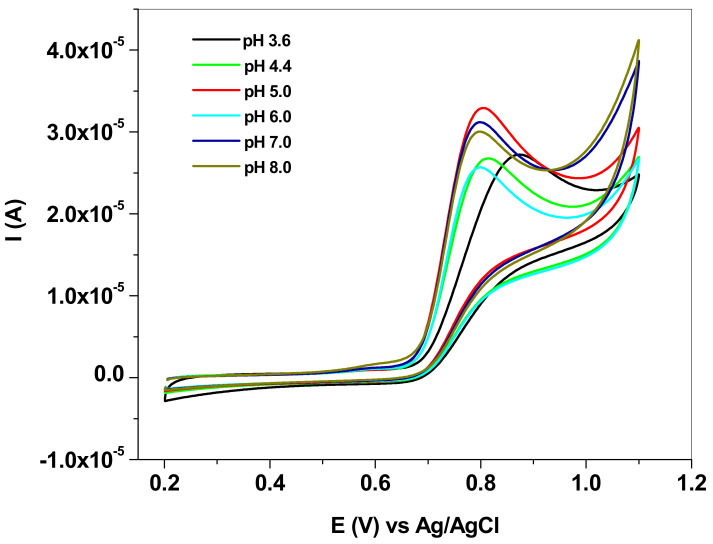
Cyclic voltammograms recorded with EGr/GC electrode in solutions of various pHs (3.6–8.0), each containing 10^−3^ M sodium nitrite (NaNO_2_); 10 mV/s scan rate.

**Figure 5:**
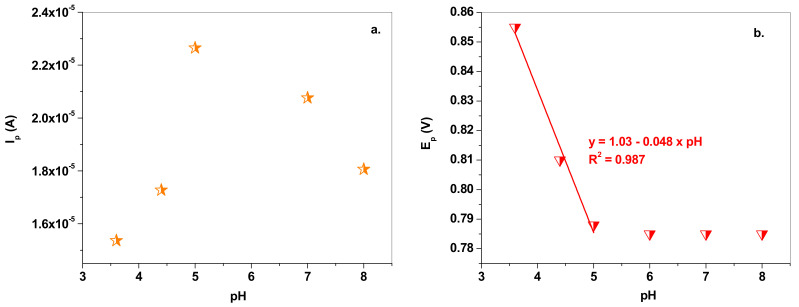
(**a**) Variation of peak current (I_p_) with the pH solution; (**b**) variation of the peak potential (E_p_) with the pH solution.

Next, the experiments were focused on studying the effect of the pH solution on the electrochemical response of the EGr/GC electrode towards NO_2_^−^ oxidation (10^−3^ M). The influence was studied by cyclic voltammetry, within the 3.6–8.0 pH range. As can be seen in Figure 4, both the peak current and the peak potential are affected by the pH. In the case of peak current (I_p_), its value increased with the increase of pH up to pH 5.0, then began to decrease (see Figure 5a). On the other hand, the peak potential (E_p_) strongly decreased in acidic solution (pH 3.6–pH 5.0), then kept an almost constant value between pH 6.0 and pH 8.0 (see Figure 5b). According to the literature, in acidic solution the nitrite anions are protonated (HNO_2_) due to their low pKa value (3.3) [46] and so protons may be involved in the electro-oxidation process. At higher pH (>5.0), the shortage of protons will make the oxidation of nitrite more difficult and, consequently, the peak current decreases. Therefore, acetate buffer of pH 5.0 was selected as the optimum electrolyte for the detection and determination of nitrite in laboratory and real solutions.

Since the NO_2_^−^ oxidation is an irreversible process (no peak in the reverse scan), the Laviron equation [47] may be used to determine the number of electrons involved in the reaction. Equation (2) shows the variation of the peak potential, E_p,_ with the natural logarithm of the scan rate, lnυ, for irreversible reactions (oxidation) when employing the linear sweep voltammetry technique.

(2) $$E_{p}=E^{0}\prime -\frac{RT}{(1-\alpha_{a})nF}\ln\frac{RTk_{s}}{(1-\alpha_{a})nF}+\frac{RT}{(1-\alpha_{a})nF}\ln\upsilon$$

where *α_a_* is the charge transfer coefficient, *k_s_* is the standard rate constant of the surface reaction, *n* is the number of electrons involved in the reaction and *E*^0^′ is the formal potential.

**Figure 6:**
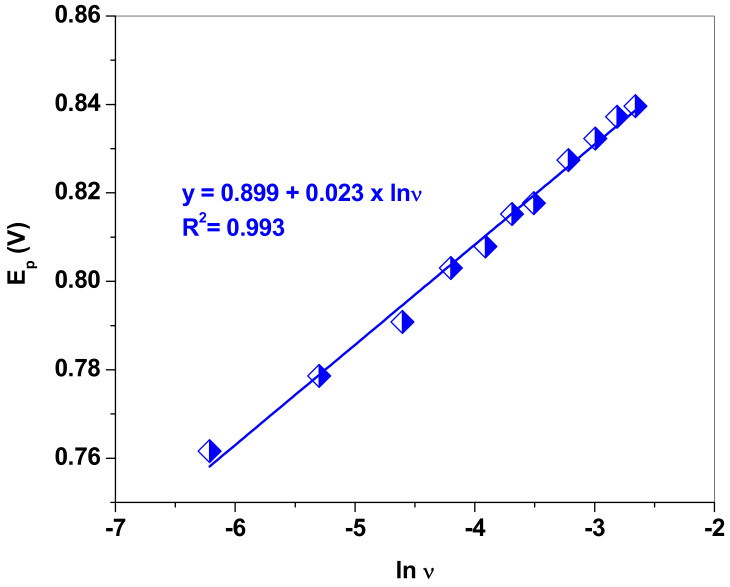
The dependence of the peak potential (E_p_) versus the natural logarithm of scan rate (lnυ) for the EGr/GC electrode.

LSVs were recorded in pH 5.0 acetate solution containing 10^−3^ M NaNO_2_ at various scanning rates, from 2 to 100 mV/s. Based on the E_p_ vs. lnυ plot and the linear regression equation (E_p_ = 0.899 + 0.023 × lnυ), we determined the value of (1−α_a_)*n* from the corresponding slope (Figure 6). Assuming that, for a totally irreversible electrode process, α_a_ is ~0.5, the number of electrons involved in the oxidation of nitrite is equal to two. As previously reported by Ma et al. [48], the electrochemical oxidation process of NO_2_^−^ at EGr/GC electrode in acidic solution follows the mechanism (Equation (3)):

NO_2_^−^ + H_2_O -------> NO_3_^−^ + 2H^+^ + 2e^−^ (3)

**Figure 7:**
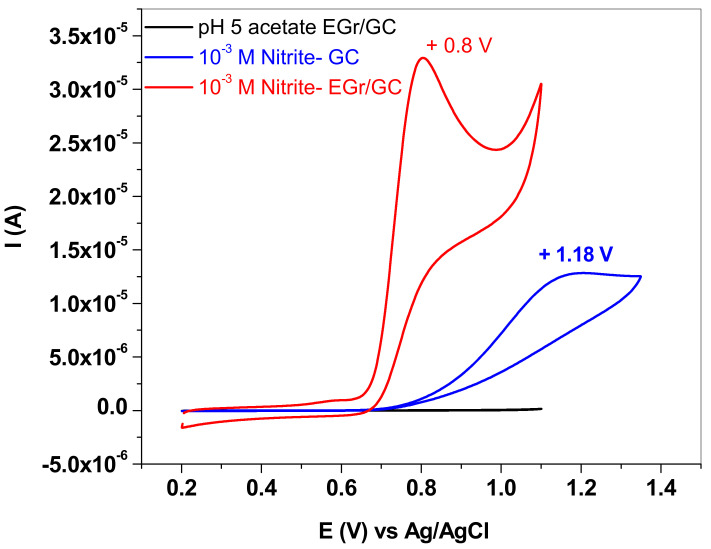
CVs recorded with bare and graphene-modified electrode (blue and red curve, respectively) in solution containing 10^−3^ M sodium nitrite in pH 5.0 acetate; 10 mV/s scan rate.

In order to evidence the electro-catalytic properties of the EGr/GC electrode towards NO_2_^−^ oxidation, CV measurements were recorded with bare and graphene-modified electrodes and the results are presented in Figure 7 (10^−3^ M NaNO_2_ in pH 5.0 acetate buffers; 10 mV/s scan rate). It is worth mentioning that, in the case of the bare GC electrode (blue curve), the oxidation peak is small and broad and appears at very high potential (+1.18 V). In contrast, the EGr/GC electrode exhibits a considerably higher electrochemical peak (three times higher) and the peak potential is shifted towards lower values (+0.8 V). Such behavior confirms that the graphene layer has an important role in promoting the electron transfer from the solution containing NO_2_^−^ anions to the glassy-carbon surface.

**Figure 8:**
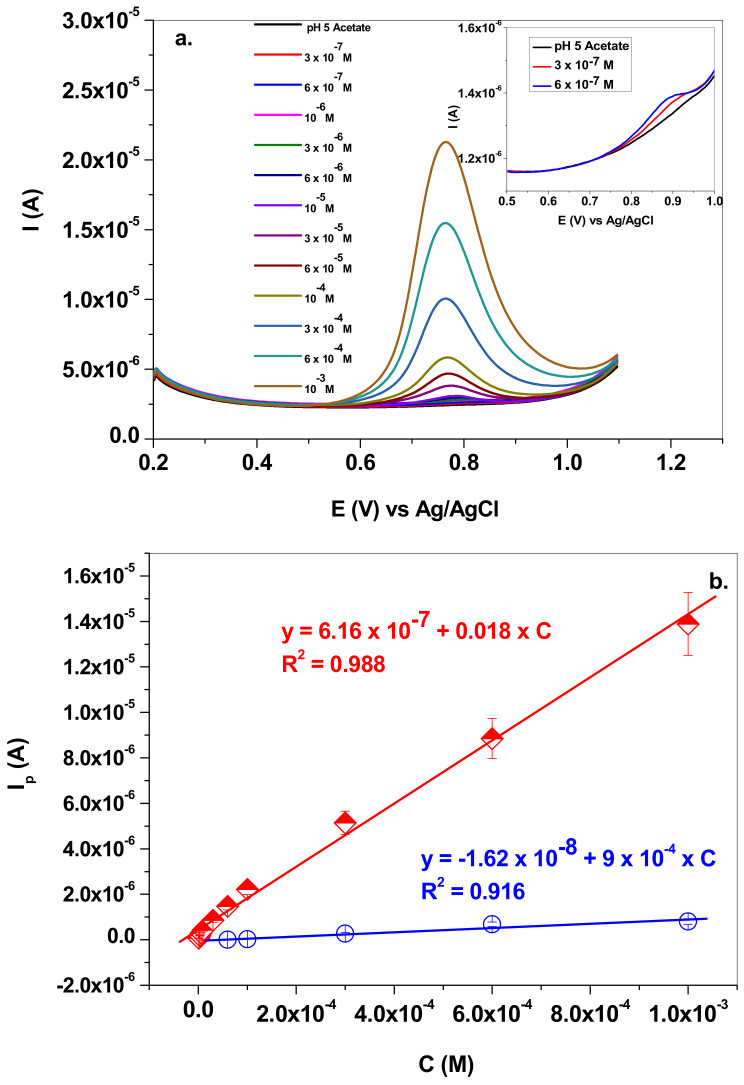
(**a**) SW voltammograms recorded with graphene-modified electrode in solutions containing various concentrations of sodium nitrite (3 × 10^−7^–10^−3^ M) in pH 5.0 acetate; 10 mV/s scan rate; (**b**) corresponding calibration plot (EGr/GC—red curve) in comparison with that obtained for bare GC electrode (blue curve).

Next, the electrochemical parameters for NO_2_^−^ detection were determined by employing the SWV technique. This technique is more sensitive in comparison with the classical cyclic voltammetry, due to the elimination of the capacitive current. Figure 8a presents the SW voltammograms recorded with a graphene-modified electrode in solutions containing various concentrations of sodium nitrite (3 × 10^−7^–10^−3^ M NaNO_2_; pH 5.0 acetate; 10 mV/s scan rate). The inset of the figure shows the signals recorded at very low concentrations (e.g., 3 × 10^−7^ and 6 × 10^−7^ M). In these cases, the peak potential is slightly shifted towards higher potentials (+0.88 V), which is a normal behavior for an irreversible process.

The corresponding calibration plot is presented in Figure 8b, in comparison with that obtained for the bare GC electrode. The linear regression equations corresponding to each electrode are: I_p_ = 6.16 × 10^−7^ + 0.018 × C (R^2^ = 0.988) for EGr/GC; and I_p_ = −1.62 × 10^−8^ + 9 × 10^−4^ × C (R^2^ = 0.916) for bare GC. For the graphene-modified electrode, the linear range was 3 × 10^−7^–10^−3^ M, the sensitivity was determined to be 0.018 A/M, the limit of quantification (LOQ) was 3 × 10^−7^ M and the limit of detection (LOD) was 9.9 × 10^−8^ M. As expected, the bare GC electrode had considerably lower performances, such as: linear range: 6 × 10^−5^–10^−3^ M; sensitivity 9 × 10^−4^ A/M; limit of quantification (LOQ) 6 × 10^−5^ M; and limit of detection (LOD) 1.82 × 10^−5^ M.

**Figure 9:**
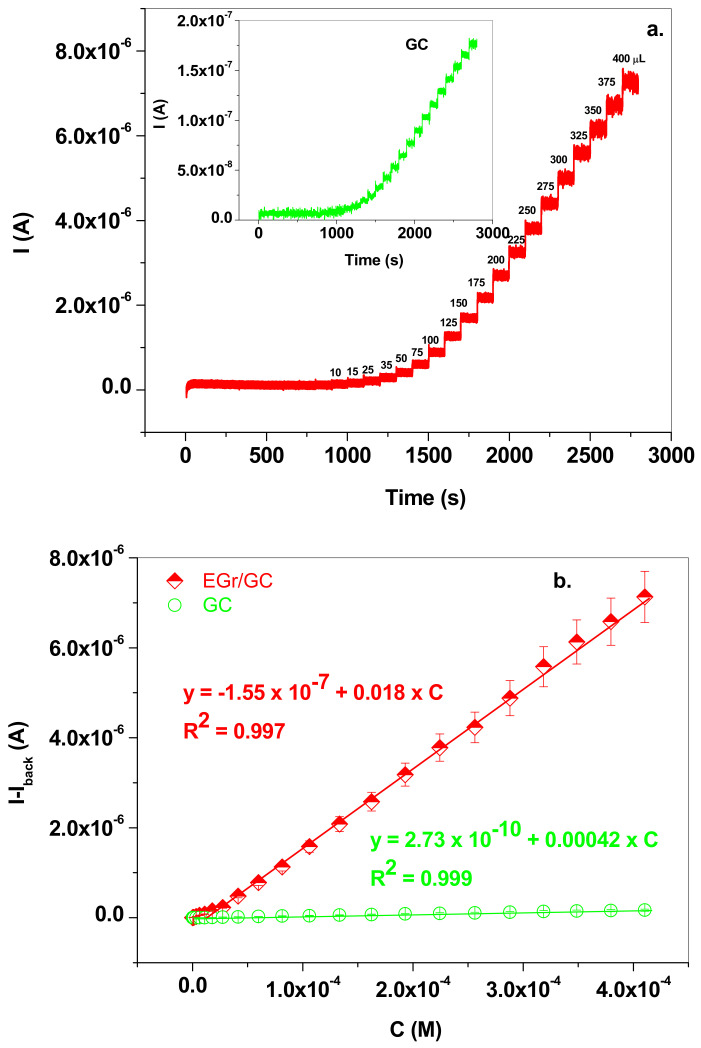
(**a**) Amperometric signals recorded with EGr/GC (red; +0.8 V applied potential) and GC (inset, green; +1.18 V applied potential) electrodes after the addition of NaNO_2_ from 10^−3^ M stock solution in supporting electrolyte of pH 5.0; the concentration range was from 3 × 10^−7^ to 4 × 10^−4^ M for EGr/GC electrode and from 3.3 × 10^−6^ to 4 × 10^−4^ M for bare GC; (**b**) The corresponding calibration curves for EGr/GC (red) and GC (green) electrodes, using background subtracted signals (**b**).

Similar results were obtained by employing the amperometric technique and the recorded signals and calibration plots are presented in Figure 9a,b. In this case, the linear range for EGr/GC electrode was also wide, from 3 × 10^−7^ to 4 × 10^−4^ M NaNO_2_, the limit of quantification (LOQ) was 3 × 10^−7^ M, the sensitivity was 0.018 A/M and the detection limit (LOD) was 9.09 × 10^−8^ M. The detection limit was calculated by dividing the limit of quantification by 3.3. For plotting of the calibration curves, we employed background subtracted signals. As observed in the case of the SW technique, the sensitivity of the EGr/GC electrode was two orders of magnitude higher than that of the bare GC, demonstrating the advantages of using graphene-modified electrodes in the electrochemical detection of nitrite ions.

The performances of the EGr/GC electrode towards nitrite detection were compared with those of other modified electrodes reported in the literature (Table 1). With few exceptions, the performances are similar, or even better, both in terms of linear range and limit of detection.

**Figure 10:**
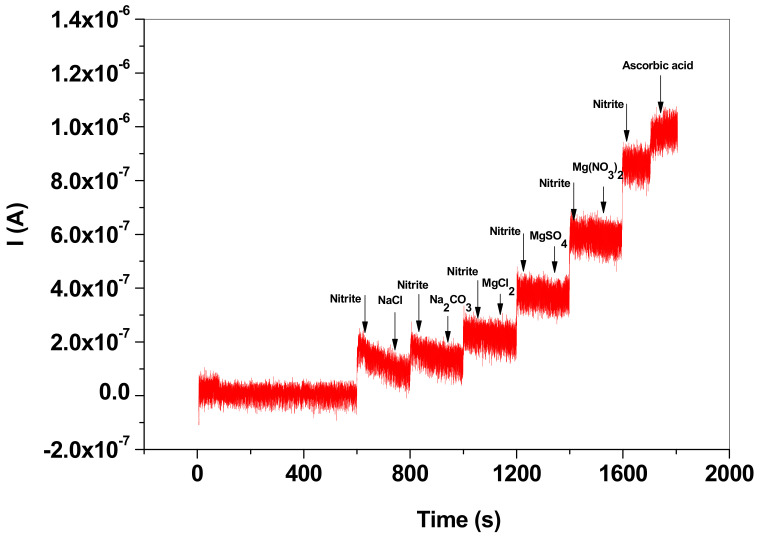
Amperometric signal recorded with EGr/GC electrode in the presence of nitrite and interfering species: NaCl, Na_2_CO_3_, MgCl_2_, MgSO_4_, Mg(NO_3_)_2_ and ascorbic acid; the interfering species had a concentration of 10^−5^ M (+0.8 V applied potential; pH 5.0 acetate buffer).

In order to prove the selectivity of the EGr/GC electrode towards NO_2_^−^ detection different interfering species were employed (Figure 10). The amperometric signal recorded at a potential of +0.8 V indicates that Na_2_CO_3_, MgCl_2_, MgSO_4_, Mg(NO_3_)_2_ had no effect on the electrochemical signal of the NO_2_^−^ specie. NaCl slightly decreased the NO_2_^−^ signal, while ascorbic acid increased the signal.

**Figure 11:**
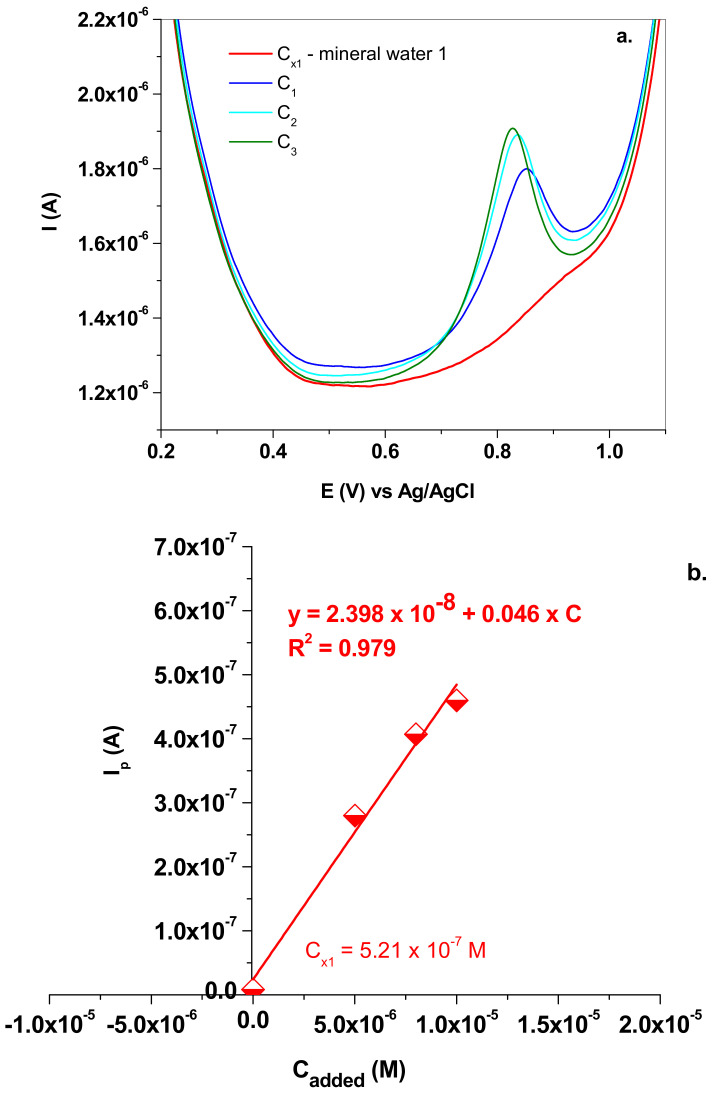
(**a**) SW voltammograms recorded in mineral water 1 (C_x1_) and in solutions containing the added NaNO_2_ (C_1_; C_2_ and C_3_); 10 mV/s scan rate; (**b**) the standard addition plot which allowed the determination of C_x1_.

Based on the fact that the electrode exhibited excellent electro-catalytic properties, it was further used for the determination of nitrite in mineral waters. Two commercial waters were employed, bought from a local supermarket. According to the label, the first one contains the minerals (mean value; mg/L): Ca^2+^ (73.65); Mg^2+^ (3.101); Na^+^ (8.655); HCO_3_ (250.1); SO_4_^−^ (<40); Cl^−^ (16.379); K^+^ (1.078); and NO_2_^−^ (<0.05). The following procedure was employed to determine the NO_2_^−^ concentration from the water. First, the water pH was brought to pH 5.0, after the addition of the corresponding amounts of acetic acid (0.2 M) and sodium acetate (0.2 M). The electrochemical signal of NO_2_^−^ was recorded in the mineral water solution and its concentration was denoted as C_x1_. Next, four beakers were filled with mineral water and increasing volumes (50; 80; and 100 µL) of NaNO_2_ stock solution (10^−3^ M). The final volume in each beaker was 10 mL and the added NaNO_2_ concentrations were denoted as C_1_, C_2_ and C_3_, respectively. The corresponding SW electrochemical signals were also recorded and the peak current was represented as a function of NaNO_2_ added concentrations (Figure 11a,b). In this case, C_x1_ was found to be 5.21 × 10^−7^ M, which corresponds to 0.023 mg/L NO_2_^−^, in excellent agreement with the mineral water label (NO_2_^−^ < 0.05 mg/L).

The same procedure was applied in the case of the second mineral water, which contained the following minerals (mean value; mg/L): Na^+^ (2); K^+^ (1.4); Mg^2+^ (2.4); Ca^2+^ (9.2); F^−^ (<0.03); Cl^−^ (<5); SO_4_^−^ (6.2); HCO_3_ (42.7); NO_2_^−^ (<0.01); NO_3_ (4.29).

**Figure 12:**
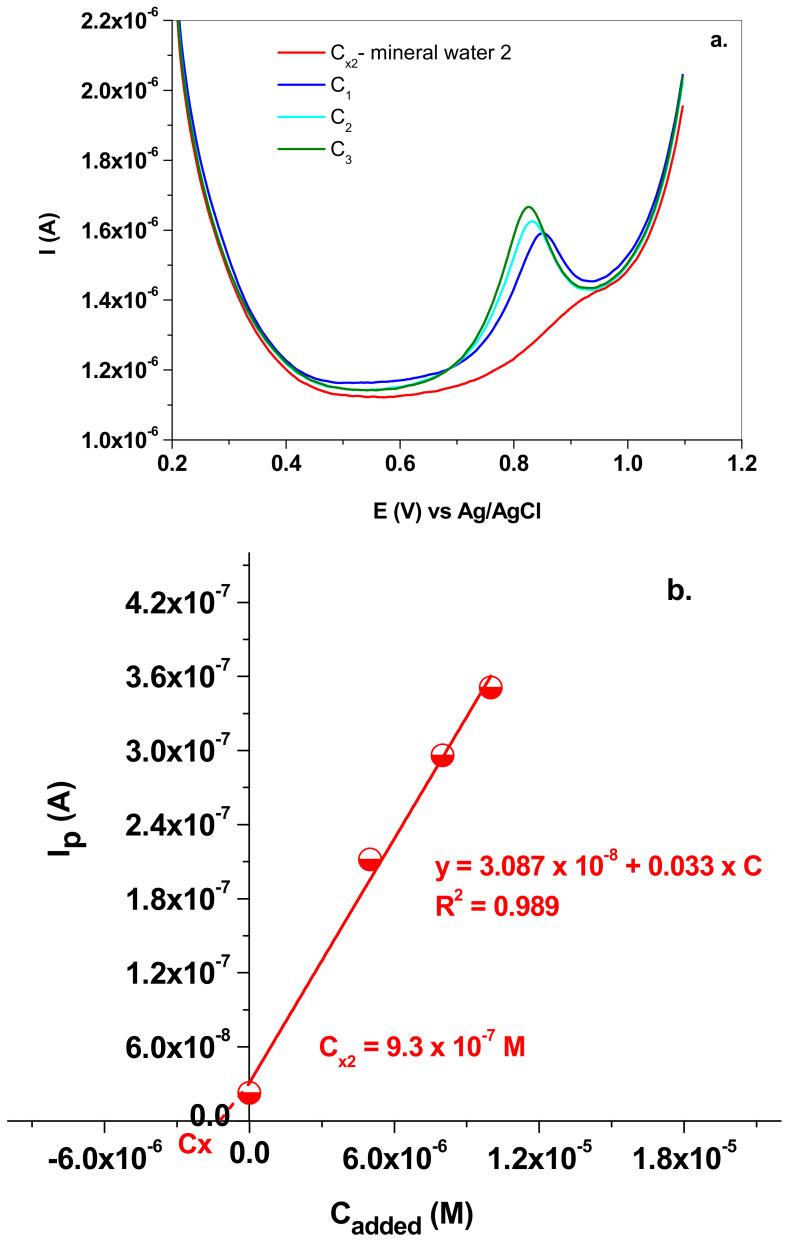
(**a**) SW voltammograms recorded in mineral water 2 (C_x2_) and in solutions containing the added NaNO_2_ (C_1_; C_2_ and C_3_); 10 mV/s scan rate; (**b**) the standard addition plot which allowed the determination of C_x2_.

The SW voltammograms can be seen in Figure 12a and the corresponding addition plot in Figure 12b. In this case, C_x2_ was determined to be 9.3 × 10^−7^ M, which corresponds to 0.04 mg/L NO_2_^−^.

To further check the accuracy of the electrochemical method, the UV-Vis colorimetric method was complementarily used. Since NaNO_2_ solution has no absorption peak in the visible range, the MQuant^®^ Nitrite Test (Merck) was employed. The test is based on the reaction between nitrite and sulphanilic acid, which forms a diazonium cation. The cation subsequently couples to the aromatic amine 1-naphthylamine and produces a red-violet azo dye with absorption at λ_max_ = 540 nm. In the case of the first mineral water, the spectrophotometric method indicated that C_x1(UV)_ = 6.21 × 10^−7^ M, while for the second mineral water C_x2(UV)_ = 7 × 10^−7^ M (Figures S3 and S4 in Supplementary Materials).

## 4. Conclusions

A graphene sample was prepared by electrochemical exfoliation of graphite rods in an electrochemical cell filled with the appropriate solution: 0.05 M (NH_4_)_2_SO_4_ + 0.1 M H_3_BO_3_ + 0.05 M NaCl. After preparation, the optimum volume (10 µL) of graphene dispersed in DMF (2 mg/mL concentration) was deposited on top of a glassy carbon electrode (EGr/GC) and then tested towards nitrite detection, in laboratory solutions and real samples. The reported results indicated that the developed graphene-based electrochemical sensor is a reliable and highly sensitive tool for nitrite assay in waters, since it displayed excellent stability and reproducibility, exhibiting a low limit of detection (9.09 × 10^−8^ M). Since the developed sensing platform is able to detect, with a high degree of accuracy and reliability low nitrite concentrations, it may be further used to monitor nitrite levels in both industrial and environmental conditions and provide a necessary tool in the prevention of water pollution.

## Figures and Tables

**Table 1:** Performance evaluation of various modified electrodes for nitrite detection.

Modified Electrode	Method	Linear Range (µM)	LOD (µM)	Reference
**Ni/PDDA/rGO/SPCE** Ni/PDDA/rGO—nickelpoly(diallyldimethylammonium chloride) reduced graphene oxide composite SPCE—screen-printed carbon electrode	CV	6–100	1.99	[27]
**LaAlO_3_@GO/GCE** LaAlO_3_@GO—La-based perovskite-type lanthanum aluminate nanorod-incorporated graphene oxide nanosheets GCE—glassy carbon electrode	CV	0.01–1540.5	0.0041	[31]
**Fe_2_O_3_/rGO/GCE** Fe_2_O_3_/rGO—hematite—reduced graphene oxide composite GCE—glassy carbon electrode	DPV	0.05–780	0.015	[29]
**GO/PEDOT:PSS/GCE** GO/PEDOT:PSS—graphene oxide—poly(3,4-ethylenedioxythiophene):poly(styrenesulfonate) (PEDOT:PSS) composite GCE—glassy carbon electrode	DPV	1–200	0.5	[49]
**AuCu NCNs/GCE** AuCu NCNs—gold-copper nanochain network GCE—glassy carbon electrode	DPV	10–4000	0.2	[33]
**CoN-CRs/GCE** CoN-CRs—Co@N-doped carbon nanorods GCE—glassy carbon electrode	AMP	0.5–8000	0.17	[30]
**Co_3_O_4_-rGO/CNTs/GCE** Co_3_O_4_-rGO/CNTs—cobalt oxide decorated reduced graphene oxide and carbon nanotubes GCE—glassy carbon electrode	AMP	0.1–8000	0.016	[32]
**Ag–Fe_3_O_4_–GO/GCE** Ag–Fe_3_O_4_–GO—Ag–Fe_3_O_4_–graphene oxide magnetic nanocomposites GCE—glassy carbon electrode	AMP	0.5–7200	0.17	[28]
**AuNPs/MoS_2_/Gr/GCE** AuNPs/MoS_2_/Gr—rose-like Au nanoparticles/MoS_2_ nanoflower/graphene composite GCE—glassy carbon electrode	AMP	5–5000	1	[50]
**Au/FePc(tBu)_4_/GCE** Au/FePc(tBu)_4_—Fe(II) tetra-tert-butyl phthalocyanine film decorated with gold nanoparticles heterostructure GCE—glassy carbon electrode	AMP	2–26 20–120	0.35	[51]
**AgNPs/GCE** AgNPs—crystalline silver nanoplates GCE—glassy carbon electrode	AMP	10–1000	1.2	[52]
**AgMC-PAA/PVA/SPCE** AgMCs-PAA/PVA—silver microcubics-polyacrylic acid/poly vinyl alcohol SPCE—screen printed carbon electrode	AMP	2–800	4.5	[53]
**AgNPs/TPDT–SiO_2_/GCE** AgNPs/TPDT–SiO_2_—silver nanoparticles (Ag NPs) deposited on amine functionalized silica (SiO2) spheres GCE—glassy carbon electrode	SWV	1–10	1	[54]
**AgPs-IL-CPE/CPE** AgPs-IL-CPE—carbon powder decorated with silver sub-micrometre particles (AgPs) and a hydrophobic ionic liquid trihexyltetradecylphosphonium chloride CPE—carbon paste electrode	SWV	50–1000	3	[55]
**EGr/GC**	AMP SWV	0.3–400 0.3–1000	0.0909	current study

## Data Availability

Data will be provided upon reasonable request to the corresponding author.

## Supplementary Materials

The following supporting information can be downloaded at: https://www.mdpi.com/article/10.3390/nano13091468/s1, Figure S1: Variation of nitrite peak current with the amount of graphene deposited on top of GC electrode (10^−3^ M NaNO_2_ solution (pH 5); 2 mg/mL graphene in DMF); Figure S2: Cyclic voltammograms recorded with various scan rates (from 2 to 100 mV/s) in solution containing 10^−3^ M potassium ferrocyanide (0.2 M KCl supporting electrolyte) (a); I_p_ versus υ^1/2^ plot, which allowed the determination of the active area (b); Figure S3: UV-Vis spectra recorded in mineral water 1 (C_x1_) and in solutions containing mineral water 1 and the added NaNO_2_ volumes of 10^−3^ M stock solution (10; 30 and 50 µL corresponding to C_1_; C_2_ and C_3_ concentrations, respectively); one spoon (75 mg) of MQuant^®^ Nitrite Test powder was added in each beaker to color the solutions (a); the standard addition plot which allowed the determination of C_x1_ (b); Figure S4: UV-Vis spectra recorded in mineral water 2 (C_x2_) and in solutions containing mineral water 2 and the added NaNO_2_ volumes of 10^−3^ M stock solution (10; 30 and 50 µL corresponding to C_1_; C_2_ and C_3_ concentrations, respectively); one spoon (75 mg) of MQuant^®^ Nitrite Test powder was added in each beaker to color the solutions (a); the standard addition plot which allowed the determination of C_x2_ (b).
