# Supplementary material for: Highly Sensitive Graphene-Based Electrochemical Sensor for Nitrite Assay in Waters
Source: Nanomaterials (Basel). 2023 Apr 25;13(9):1468. doi: 10.3390/nano13091468 (PMC10179868; doi:10.3390/nano13091468)
Supplement: Supplementary file 1 [file nanomaterials-13-01468-s001.zip › nanomaterials-2343221-supplementary.pdf]

Supporting Information

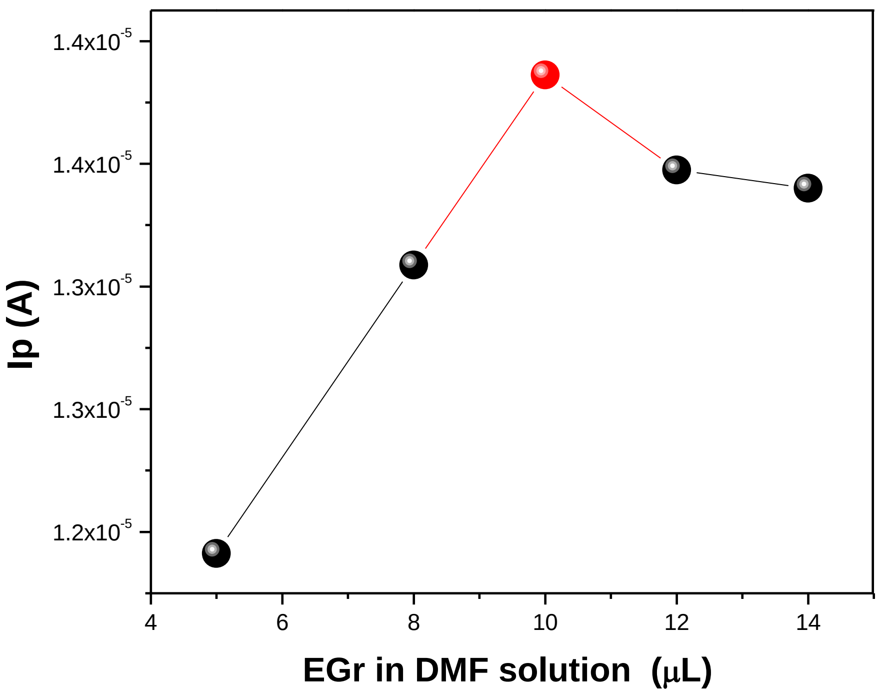

Figure S1. Variation of nitrite peak current with the amount of graphene deposited on top of GC electrode (10^{-3} M $NaNO_2$ solution (pH 5.0); 2 mg/mL graphene in DMF)

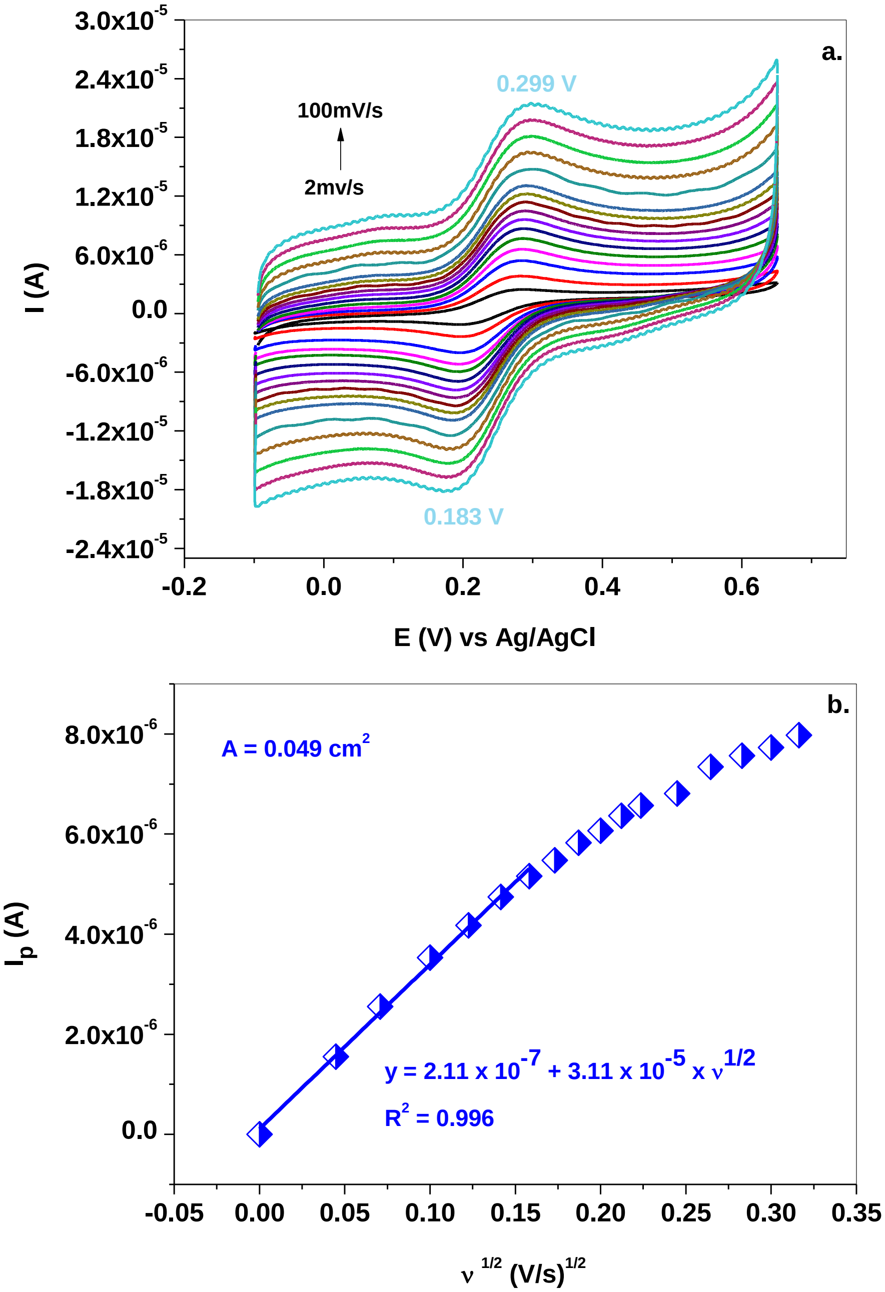

Figure S2. Cyclic voltammograms recorded with various scan rates (from 2 to 100 mV/s) in solution containing 10^{-3} M potassium ferrocynide (0.2 M KCl supporting electrolyte) (a);

I_P *versus* $\upsilon^{1/2}$ plot, which allowed the determination of the active area (b)

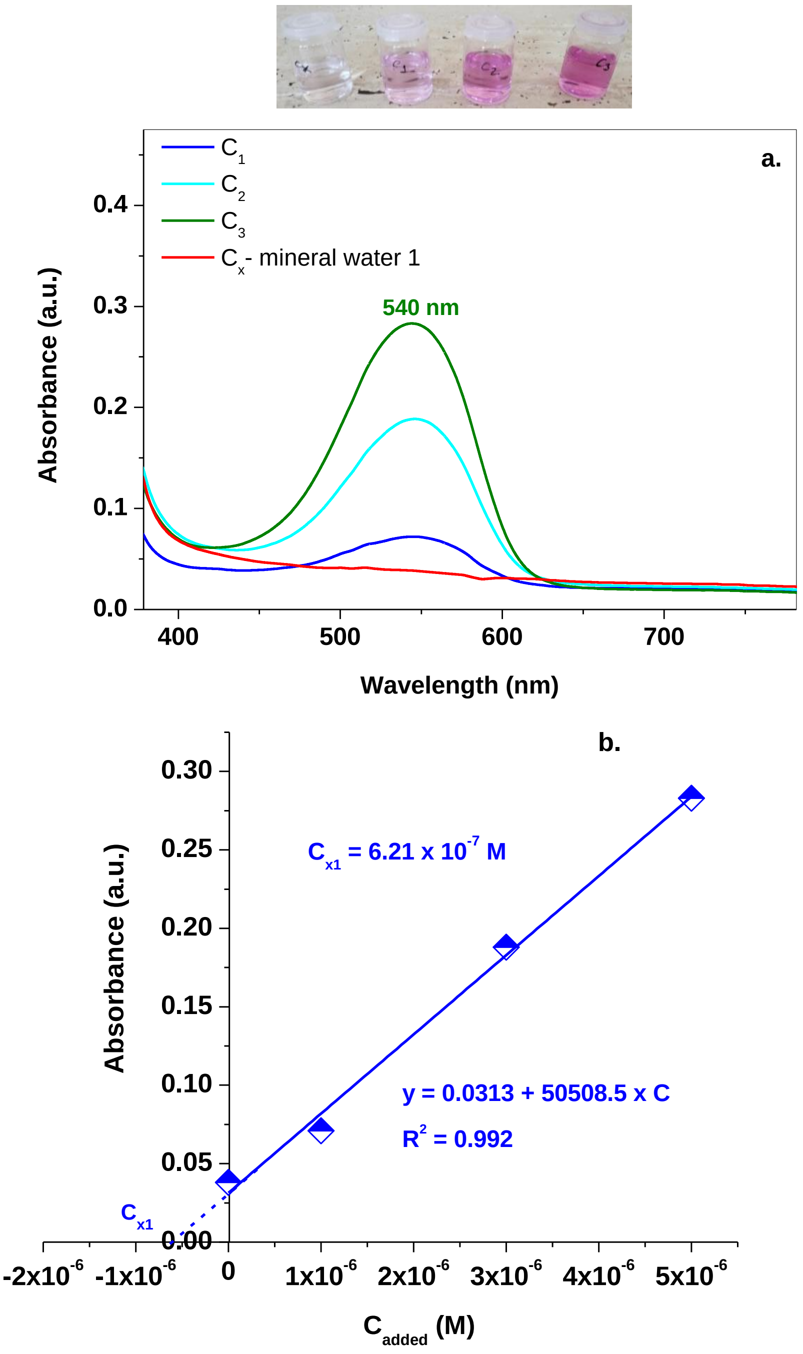

Figure S3. UV-Vis spectra recorded in mineral water 1 (C_{x1}) and in solutions containing mineral water 1 and the added $NaNO_2$ volumes of 10^{-3} M stock solution (10; 30 and 50 μL corresponding to C_1; C_2 and C_3 concentrations, respectively); one spoon (75 mg) of MQuant® Nitrite Test powder was added in each beaker to color the solutions (a); the standard addition plot which allowed the determination of C_{x1} (b)

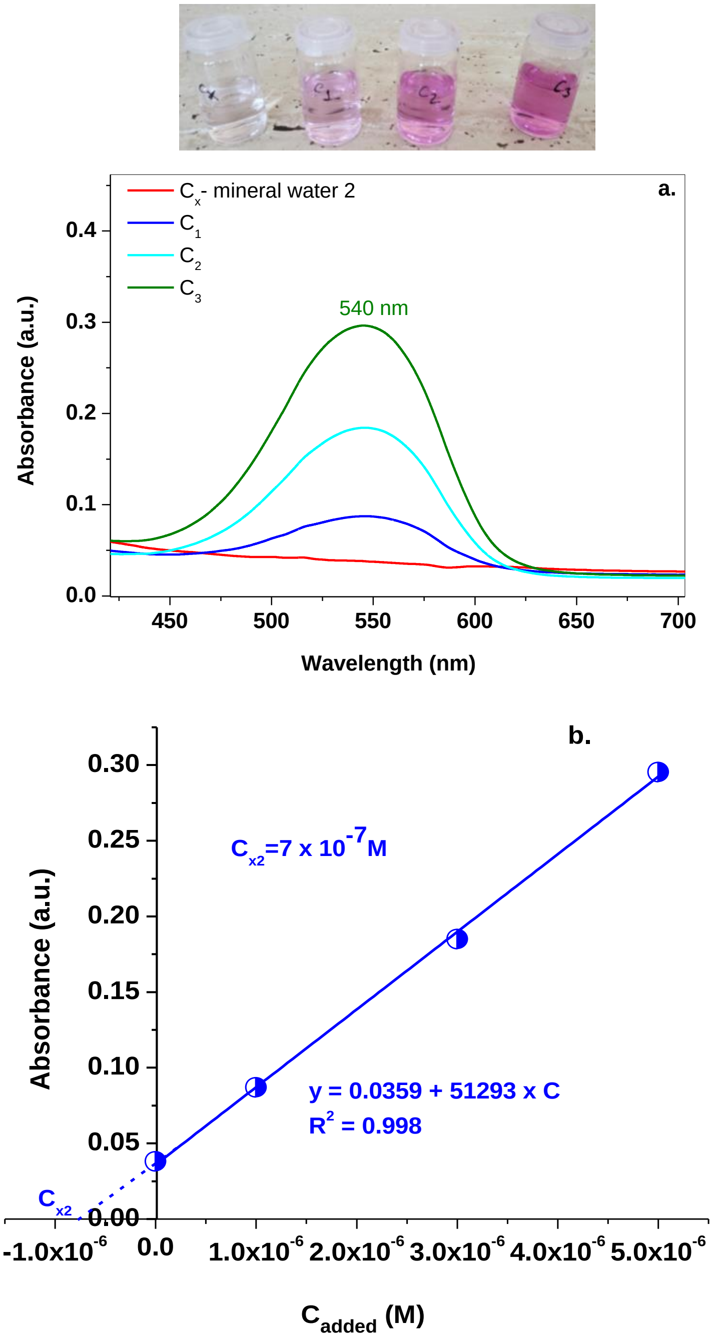

Figure S4. UV-Vis spectra recorded in mineral water 2 (C_{x2}) and in solutions containing mineral water 2 and the added $NaNO_2$ volumes of 10^{-3} M stock solution (10; 30 and 50 µL corresponding to C_1; C_2 and C_3 concentrations, respectively); one spoon (75 mg) of MQuant® Nitrite Test powder was added in each beaker to color the solutions (a); the standard addition plot which allowed the determination of C_{x2} (b)
